# Supplementary material for: Characterizing changes in behaviors associated with chemical exposures during the COVID-19 pandemic
Source: PLoS One. 2023 Jan 13;18(1):e0277679. doi: 10.1371/journal.pone.0277679 (PMC9838870; doi:10.1371/journal.pone.0277679)
Supplement: S1 File — (PDF) [file pone.0277679.s001.pdf]

COHORT ID	SITE ID	PARTICIPANT ID	PIN	COHORT VISIT ID	FORM COMPLETED
__ __ __ __ __ __	__ __ __ __	____________	__ __ __	________	__ __/__ __/__ __ __ __ mm dd yyyy

ECHO LIFE STAGE		RESPONDENT	
☐01 Prenatal	☐02 Perinatal	☐01 Participant	☐02 Biological Mother
☐03 Infancy	☐04 Early Childhood	☐03 Biological Father	☐04 Other Respondent → Code: __ __
☐05 Middle Childhood	☐06 Adolescence		

STUDY STAFF INSTRUCTION: This form should be completed by the pregnant woman enrolled in an ECHO cohort during the prenatal life stage and by the primary caregiver of a child enrolled in an ECHO cohort during any other life stage. In the prenatal life stage, the woman's pregnancy ID should be used in the header for the participant ID. In all other life stages, the child's ID should be used in the header for the participant ID.

INSTRUCTIONS:
These questions are about your experience with COVID-19, or the coronavirus. For each question, do the best you can to remember the details requested, as many ask you to remember what you did before March 2020 (prior to the pandemic).

There are 8 sections in this survey:

- Section A: Current Circumstances
- Section B: Food
- Section C: Personal Care Products
- Section D: Consumer Products
- Section E: Kid Products
- Section F: Housing
- Section G: Time Spent Indoors
- Section H: Transportation

Section A. Current Circumstances

1. Which of the following situations currently applies to the child's household? ***[Mark all that apply]***

☐01 Local government ordered residents to stay at home
☐02 Local government encouraged residents to stay at home
☐03 Local government made social distancing recommendations, but did not encourage or order residents to stay at home
☐04 Local government did not make stay at home or social distancing recommendations

2. Which best describes practices that members of the child's household are currently following?

☐01 All members of the household have decided to stay at home ➔ ***Skip to Section B, question 1***
☐02 Some members of the household have decided to stay at home and some have decided not to stay home ➔ ***If marked, answer Section A, question 2.a***
☐03 No restrictions on whether members of the household stay at home ➔ ***Skip to Section B, question 1***

2.a. What are the reasons that some of the members of the household do not stay at home ***[Mark all that apply]***

☐01 Work requirements (e.g., essential workers)
☐02 Essential shopping needs (e.g., buying food)
☐03 Medical needs (e.g., doctor visits)
☐04 Social needs (e.g., visited friends or family members)
☐05 Educational needs (e.g., children attending school, even part time)
☐06 Other, specify ______________________

Section B. Food

These next questions are about the food eaten in your household in the last month compared to the same month, prior to the beginning of the COVID-19 pandemic.

1. Compared to this time of year in 2019, did you eat more/less/about the same amount of **fruits or vegetables?**

	Less	About the same	More
1.a. **Fresh** fruits or vegetables	☐01	☐02	☐03
1.b. **Canned** fruits or vegetables	☐01	☐02	☐03
1.c. **Frozen** fruits or vegetables	☐01	☐02	☐03
1.d. **Dried** fruits or vegetables	☐01	☐02	☐03
1.e. **Organic** fruits or vegetables	☐01	☐02	☐03

2. Compared to this time of year in 2019, did you eat more/less/about the same amount of **the following food groups?**

	Less	About the same	More
2.a. **Dairy** products	☐01	☐02	☐03
2.b. **Meat** products	☐01	☐02	☐03
2.c. **Fish or seafood** purchased from a store or restaurant	☐01	☐02	☐03
2.d. **Fish or seafood** caught from a river, lake, or stream	☐01	☐02	☐03

3. Compared to this time of year in 2019, did you eat more/less/about the same amount of **the following types of foods?**

	Less	About the same	More
3.a. **Canned** foods (other than fruits and vegetables)	☐01	☐02	☐03
3.b. **Fast food** (e.g., McDonalds, KFC, Subway, Chipotle)	☐01	☐02	☐03
3.c. **Meals cooked or prepared at home**	☐01	☐02	☐03
3.d. Foods that are **prepared or ready-to-eat** (e.g., pizza, sandwiches, soups)	☐01	☐02	☐03

4. Compared to this time of year in 2019, did you eat more/less/about the same amount of **the following?**

	Less	About the same	More
4.a. **Sweetened milk substitute** (e.g., soy milk, almond milk)	☐01	☐02	☐03
4.b. **Sweetened yogurt or ice cream**	☐01	☐02	☐03
4.c. **Sweetened beverages (e.g., juice drinks, sodas)**	☐01	☐02	☐03
4.d. **Alcoholic beverages**	☐01	☐02	☐03
4.e. **Commercially made desserts** (e.g., pudding, flan)	☐01	☐02	☐03
4.f. **Frozen meats** (e.g., chicken nuggets, fish sticks, hotdogs, sausage)	☐01	☐02	☐03
4.g. **Meatless patties**	☐01	☐02	☐03
4.h. **Commercially-made breads** (including tortillas, instant noodles)	☐01	☐02	☐03
4.i. **Dry cake mixes** (including cookies, brownies)	☐01	☐02	☐03
4.j. **Packaged snacks** (e.g., chips, bars, pretzels)	☐01	☐02	☐03
4.k. **Sweetened breakfast cereals**	☐01	☐02	☐03

Section B. Food (continued)

5. Compared to this time of year in 2019, did you drink more/less/about the same amount of **the following beverages?** Include beverages poured from a can, bottle, or carton into a glass before drinking.

	Less	About the same	More
**5.a.** **Canned** beverages (e.g., soda, seltzer, beer)	☐01	☐02	☐03
**5.b.** Plastic **bottled** beverages (e.g., soda, juice, sparkling water)	☐01	☐02	☐03
**5.c.** Beverages from a **carton or pouch** (e.g., milk, capri sun)	☐01	☐02	☐03

6. **Since the COVID-19 pandemic began**, did you move or change water **systems** (e.g., from public water to private well or vice versa)?

☐01 Yes
☐02 No ➔ ***Skip to Section C, question 1***
☐03 Not sure ➔ ***Skip to Section C, question 1***

7. Compared to this time of year in 2019, did you drink more/less/about the same amount of **water from the following sources** (do not include store bought seltzer or flavored water)?

	Less	About the same	More
**7.a.** **Filtered tap water**	☐01	☐02	☐03
**7.b.** **Tap water** (without it going through a filter)	☐01	☐02	☐03
**7.c.** **Bottled water**	☐01	☐02	☐03
**7.d** **Local Spring** (well)	☐01	☐02	☐03
**7.e** **Other source** (e.g., work, restaurant, etc.)	☐01	☐02	☐03

Section C. Personal Care Products

These next questions are about the personal care products that you have used in the last month compared to the same month, prior to the beginning of the of the COVID-19 pandemic.

1. Compared to this time of year in 2019, did you use more/less/about the same amount of **the following personal care products?**

	Less	About the same	More
**1.a.** **Perfume**	☐01	☐02	☐03
**1.b.** **Deodorant**	☐01	☐02	☐03
**1.c.** **Lotion**	☐01	☐02	☐03
**1.d** **Makeup**	☐01	☐02	☐03
**1.e.** **Nail polish** (include polish applied to fingers OR toes)	☐01	☐02	☐03
**1.f.** **Sunscreen**	☐01	☐02	☐03

2. Compared to this time of year in 2019, did you use more/less/about the same amount of **the following hand/body cleaners?**

	Less	About the same	More
**2.a.** **Liquid Soap** (include both antibacterial and non-antibacterial)	☐01	☐02	☐03
**2.b.** **Antibacterial Soap**	☐01	☐02	☐03
**2.c.** **Waterless hand sanitizer gels (or liquid)**	☐01	☐02	☐03

Section C. Personal Care Products (continued)

3. Compared to this time of year in 2019, did you use more/less/about the same amount of **the following hair products?**

	Less	About the same	More
**3.a. Hair Gel**	☐01	☐02	☐03
**3.b. Hair Spray**	☐01	☐02	☐03
**3.c. Hair dye** (include both store bought and from salon)	☐01	☐02	☐03
**3.d. Perms or relaxers** (include both home and salon treatments)	☐01	☐02	☐03

Section D. Consumer Products

The next question is about the cleaning products that you have used in your household the last month compared to the same month, prior to the beginning of the COVID-19 pandemic.

1. Compared to this time of year in 2019, did you use more/less/about the same amount of **the following house cleaning products?**

	Less	About the same	More
**1.a. Antibacterial cleaners or wipes** (e.g., Lysol)	☐01	☐02	☐03
**1.b. Mold or mildew cleaners**	☐01	☐02	☐03
**1.c. Cleaning products or wipes with bleach** (e.g., Clorox)	☐01	☐02	☐03
**1.d. Cleaning products with fragrance**	☐01	☐02	☐03

2. Is your child that is currently enrolled in ECHO between the ages of 1 and 5 years old?
   ☐01 Yes
   ☐02 No ➔ ***Skip to Section F, question 1***

Section E. Kid Products

The next question is about the personal care products that your child has used in the last month compared to the same month, prior to the beginning of the COVID-19 pandemic.

1. Compared to this time of year in 2019, did you use more/less/about the same amount of the **following products on your child?**

	Less	About the same	More
**1.a. Shampoo** (including baby shampoo)	☐01	☐02	☐03
**1.b. Baby wash**	☐01	☐02	☐03
**1.c. Conditioner**	☐01	☐02	☐03
**1.d. Lotion** (including baby lotion)	☐01	☐02	☐03
**1.e. Bubble bath**	☐01	☐02	☐03
**1.f. Liquid soap** (include both antibacterial and non-antibacterial)	☐01	☐02	☐03
**1.g. Waterless hand sanitizer gel or liquid**	☐01	☐02	☐03
**1.h. Nail polish**	☐01	☐02	☐03
**1.i. Sunscreen**	☐01	☐02	☐03

Section F. Housing

1. Have **you moved or changed the address where you spend the majority of your time** since March 2020?
   ☐01 Yes ➔ ***Skip to Section F, question 2***
   ☐02 No

   1.a. Compared to this time of year in 2019, did you feel there is more/less/about the same amount of traffic on the road closest to your home?
   ☐01 More traffic
   ☐02 About the same amount of traffic
   ☐03 Less traffic

2. How many adults (including yourself) live in the household where you currently spend the majority of your time?
   |__|__| Number of adults

3. How many children live in the household where you currently spend the majority of your time?
   |__|__| Number of children

4. How many **rooms** (not including the kitchen or bathrooms) do you have in the household where you currently spend the majority of your time?
   |__|__| Number of rooms

5. When you think about the room(s) in your home where you currently spend the most time while you are awake, do you usually keep the window(s) or doors that face outside:
   ☐01 Completely closed
   ☐02 Open just a crack
   ☐03 Open partway
   ☐04 All the way open
   ☐-8 I don't know

Section G. Time Spent Indoors

These next questions are about where you have spent time in the last month compared to the same month, prior to the beginning of the COVID-19 pandemic.

1. In the past month, **on a typical weekday** (Total at the bottom should equal 24 hours—don't forget time asleep), how many hours of your time do you spend:
   1.a. Indoors at home: .... |__|__| Hours
   1.b. Indoors at work: .... |__|__| Hours
   1.c. Indoors (other): .... |__|__| Hours
   1.d. Outdoors at/near home (including balconies, patios, etc.): .... |__|__| Hours
   1.e. Outdoors at work: .... |__|__| Hours
   1.f. Outdoors (others) .... |__|__| Hours
   1.g. In transit: .... |__|__| Hours
   1.h. **Total:** .... |__|__| Hours
   1.i. **Not** sure: .... ☐-8

Section G. Time Spent Indoors (continued)

Remember, these questions are about where you have spent time in the last month compared to the same month, prior to the beginning of the COVID-19 pandemic.

2. Compared to this time of year in 2019, **on a** typical **weekday** did you spend more/the same/less time?

	More time	Same amount of time	Less time
**2.a.** Indoors at home	☐01	☐02	☐03
**2.b.** Indoors at work	☐01	☐02	☐03
**2.c.** Indoors (other)	☐01	☐02	☐03
**2.d.** Outdoors at/near home	☐01	☐02	☐03
**2.e.** Outdoors (other)	☐01	☐02	☐03
**2.f.** In transit	☐01	☐02	☐03

3. In the past month, **on a typical weekend day** (Total at the bottom should equal 24 hours—don't forget time asleep), how many hours of your time do you spend:

3.a. Indoors at home: ....|__|__| Hours
3.b. Indoors at work: ....|__|__| Hours
3.c. Indoors (other): ....|__|__| Hours
3.d. Outdoors at/near home (including balconies, patios, etc.): ....|__|__| Hours
3.e. Outdoors at work: ....|__|__| Hours
3.f. Outdoors (others) ....|__|__| Hours
3.g. In transit: ....|__|__| Hours
3.h. **Total:** ....|__|__| Hours
3.i. Not sure: .... ☐-8

4. Compared to this time of year in 2019, **on a typical weekend day** did you spend more/the same/less time?

	More time	Same amount of time	Less time
**4.a.** Indoors at home	☐01	☐02	☐03
**4.b.** Indoors at work	☐01	☐02	☐03
**4.c.** Indoors (other)	☐01	☐02	☐03
**4.d.** Outdoors at/near home	☐01	☐02	☐03
**4.e.** Outdoors (other)	☐01	☐02	☐03
**4.f.** In transit	☐01	☐02	☐03

5. In the winter, how do you heat your home (***Mark all that apply***)?

- ☐01 Radiator
- ☐02 Electric heater
- ☐03 Fireplace
- ☐04 Wood stove
- ☐05 Gas heater
- ☐06 Other, specify: ______________________
- ☐07 No heat

Section G. Time Spent Indoors (continued)

6. Do you, someone you live with, or someone who visits you regularly currently smoke tobacco inside your home?

- ☐01 Yes
- ☐02 No ➔ ***Skip to Section G, question 7***
- ☐-8 Not sure ➔ ***Skip to Section G, question 7***

6.a. Compared to this time of year in 2019, do they smoke more/about the same/less tobacco products?

- ☐01 More
- ☐02 About the same
- ☐03 Less
- ☐-6 Not applicable - they were not in my home at this time last year

7. Do you, someone you live with, or someone who visits you regularly currently smoke other non-tobacco products (such as e-cigarettes, vape, cannabis, etc) inside your home?

- ☐01 Yes
- ☐02 No ➔ ***Skip to Section H, question 1***
- ☐-8 Not sure ➔ ***Skip to Section H, question 1***

7.a. Compared to this time of year in 2019, do they smoke more/about the same/less non-tobacco products?

- ☐01 More
- ☐02 About the same
- ☐03 Less
- ☐-6 Not applicable - they were not in my home at this time last year

Section H. Transportation

These last questions are about the transportation choices that you have used in the last month compared to the same month, prior to the beginning of the COVID-19 pandemic.

1. In the last month, what **mode of transportation** did you use the most?

- ☐01 Private vehicle that you drive
- ☐02 Private vehicle that a friend or family member drives
- ☐03 Private vehicle that you hire (e.g., taxi, UBER)
- ☐04 Walk
- ☐05 Bike
- ☐06 Public transit (e.g., bus, train)
- ☐07 None
- ☐-8 I don't know

2. Compared to this time of year in 2019, what **mode of transportation** did you use the most?

- ☐01 Private vehicle that you drive
- ☐02 Private vehicle that someone else drives (e.g., taxi, UBER)
- ☐03 Walk
- ☐04 Bike
- ☐05 Public transit (e.g., bus, train)
- ☐06 None
- ☐-8 I don't know

Setting	Mode
☐01 Clinic or site ☐02 Phone ☐03 Other location	☐01 Self-administered ☐02 Staff-administered
